# Human metapneumovirus infection is associated with a substantial morbidity and mortality burden in adult inpatients

**DOI:** 10.1016/j.heliyon.2024.e33231

**Published:** 2024-06-18

**Authors:** Quentin Philippot, Blandine Rammaert, Gaëlle Dauriat, Cédric Daubin, Frédéric Schlemmer, Adrien Costantini, Yacine Tandjaoui-Lambiotte, Mathilde Neuville, Emmanuelle Desrochettes, Alexis Ferré, Laetitia Bodet Contentin, François-Xavier Lescure, Bruno Megarbane, Antoine Belle, Jean Dellamonica, Sylvain Jaffuel, Jean-Luc Meynard, Jonathan Messika, Nicolas Lau, Nicolas Terzi, Isabelle Runge, Olivier Sanchez, Benjamin Zuber, Emmanuel Guerot, Anahita Rouze, Patricia Pavese, François Bénézit, Jean-Pierre Quenot, Xavier Souloy, Anne Lyse Fanton, David Boutoille, Vincent Bunel, Astrid Vabret, Jacques Gaillat, Anne Bergeron, Nathanaël Lapidus, Muriel Fartoukh, Guillaume Voiriot

**Affiliations:** a Sorbonne Université, Assistance Publique - Hôpitaux de Paris, Service de Médecine Intensive Réanimation, Hôpital Tenon, Paris, France; b Maladies infectieuses et tropicales, CHU de Poitiers, France; c Service de Pneumologie B, Hôpital Bichat, Paris, France; d CHU de Caen Normandie, médecine intensive réanimation, 14000, CAEN, France; e Université Paris Est Créteil, Faculté de Santé, INSERM, IMRB, Créteil, France; f AP-HP, Hôpitaux Universitaires Henri Mondor, Unité de Pneumologie, Service de Médecine Intensive et Réanimation, Créteil, France; g Service de Pneumologie, APHP, Hôpital Saint Louis, France; h Service de médecine intensive réanimation, AP-HP, Hôpital Avicenne, France; i Service de médecine intensive réanimation, AP-HP, Hôpital Bichat Claude-Bernard, France; j Service de médecine intensive réanimation, AP-HP, Hôpital Saint Antoine, France; k Service de réanimation médico-chirurgicale, centre hospitalier de Versailles, France; l Médecine Intensive Réanimation, INSERM CIC 1415, CRICS-TriGGERSep Network, CHRU de Tours and methodS in Patient-Centered Outcomes and Health ResEarch (SPHERE), INSERM UMR 1246, Université de Tours, Tours, France; m Maladies infectieuses et tropicales, APHP, Hôpital Bichat Claude Bernard, France; n Service de médecine intensive réanimation, AP-HP, Hôpital Lariboisière, France; o Service de pneumologie, centre hospitalier intercommunal Compiègne Moyon, France; p Service de médecine intensive réanimation, UR2CA - Université Cote d’Azur, CHU de Nice, France; q Service de maladies infectieuses et tropicales, CHRU de Brest, France; r Maladies infectieuses et tropicales, AP-HP, Hôpital Saint Antoine, France; s Réanimation médico-chirurgicale, AP-HP, Hôpital Louis Mourier, France; t Réanimation, surveillance continue, Site de Longjumeau Groupe Hospitalier Nord-Essone, France; u Médecine Intensive Réanimation, CHU Grenoble Alpes, France; v Médecine intensive réanimation, CHR d’Orléans, France; w Université Paris Cité, Service de pneumologie et soins Intensifs, HEGP, AP-HP Centre Université Paris Cité, France; x Réanimation polyvalente, hôpital Foch, France; y Service de médecine intensive réanimation, AP-HP, HEGP, France; z Univ. Lille, Inserm U1285, CHU Lille, Service de Médecine Intensive – Réanimation, CNRS, UMR 8576, UGSF - Unité de Glycobiologie Structurale et Fonctionnelle, F-59000, Lille, France; aa Service des maladies infectieuses, CHU Grenoble Alpes, France; ab Service de Maladies Infectieuses et Réanimation Médicale, CHU de Rennes, France; ac Service de médecine intensive réanimation, CHU de Dijon, France; ad Réanimation polyvalente, Centre hospitalier public du Cotentin, France; ae Service de pneumologie et soins intensifs respiratoires, CHU Dijon Bourgogne, France; af Service de maladies infectieuses et tropicales, CHU de Nantes, France; ag FéNoMIH, CHU de Caen et de Rouen, GRAM EA2656, laboratoire de virologie, Normandie université, CHU de Caen, France; ah Service des maladies infectieuses, Hôpital d'Annecy, France; ai Service de pneumologie, Hôpitaux universitaires de Genève, Genève, Switzerland; aj Sorbonne Université, INSERM, Institut Pierre Louis d'Epidémiologie et de Santé Publique IPLESP, Public Health Department, Hôpital Saint-Antoine, Assistance Publique-Hôpitaux de Paris, Paris, France; ak Sorbonne Université, Groupe de Recherche Clinique CARMAS Université Paris Est Créteil, Assistance Publique - Hôpitaux de Paris, Service de Médecine Intensive Réanimation, Hôpital Tenon, Paris, France; al Sorbonne Université, Centre de Recherche Saint-Antoine UMRS_938 INSERM, Assistance Publique – Hôpitaux de Paris, Service de Médecine Intensive Réanimation, Hôpital Tenon, Paris, France

**Keywords:** Human metapneumovirus, Pneumonia, Viral pneumonia, Respiratory viruses

## Abstract

**Background:**

Human metapneumovirus (hMPV) is one of the leading respiratory viruses. This prospective observational study aimed to describe the clinical features and the outcomes of hMPV-associated lower respiratory tract infections in adult inpatients.

**Methods:**

Consecutive adult patients admitted to one of the 31 participating centers with an acute lower respiratory tract infection and a respiratory multiplex PCR positive for hMPV were included. A primary composite end point of complicated course (hospital death and/or the need for invasive mechanical ventilation) was used.

**Results:**

Between March 2018 and May 2019, 208 patients were included. The median age was 74 [62–84] years. Ninety-seven (47 %) patients were men, 187 (90 %) had at least one coexisting illness, and 67 (31 %) were immunocompromised. Median time between first symptoms and hospital admission was 3 [2–7] days. The two most frequent symptoms were dyspnea (86 %) and cough (85 %). The three most frequent clinical diagnoses were pneumonia (42 %), acute bronchitis (20 %) and acute exacerbation of chronic obstructive pulmonary disease (16 %). Among the 52 (25 %) patients who had a lung CT-scan, the most frequent abnormality was ground glass opacity (41 %). While over four-fifths of patients (81 %) received empirical antibiotic therapy, a bacterial coinfection was diagnosed in 61 (29 %) patients. Mixed flora (16 %) and enterobacteria (5 %) were the predominant documentations. The composite criterion of complicated course was assessable in 202 (97 %) patients, and present in 37 (18 %) of them. In the subpopulation of pneumonia patients (42 %), we observed a more complicated course in those with a bacterial coinfection (8/24, 33 %) as compared to those without (5/60, 8 %) (p = 0.02). Sixty (29 %) patients were admitted to the intensive care unit. Among them, 23 (38 %) patients required invasive mechanical ventilation. In multivariable analysis, tachycardia and alteration of consciousness were identified as risk factors for complicated course.

**Conclusion:**

hMPV-associated lower respiratory tract infections in adult inpatients mostly involved elderly people with pre-existing conditions. Bacterial coinfection was present in nearly 30 % of the patients. The need for mechanical ventilation and/or the hospital death were observed in almost 20 % of the patients.

## 1 Introduction

Respiratory virus, including influenza and non-influenza viruses, are the most common pathogens documented in hospitalized patients suffering from community-acquired acute lower respiratory tract infection [1]. Among them, the human metapneumovirus (hMPV) is found in 3–8 % of cases [[1], [2], [3]]. hMPV belongs to the pneumoviridae and was first described in 2001 [4]. hMPV is genetically close to the respiratory syncytial virus (RSV) [4]. hMPV infections have a seasonal epidemic pattern, in late fall and early winter. The incubation period varies between four and six days [5]. Clinical symptoms are an influenza-like illness associated with signs of upper and/or acute lower respiratory tract infection. Life threatening hMPV infections have been described with admission to intensive care unit (ICU) of up to 30 % of hospitalized adults with hMPV infection [6]. Current therapeutic management is mainly symptomatic. However, several drug candidates have shown an activity on hMPV, and some additional targets have recently been identified [[7], [8], [9], [10]]. As specific antiviral therapies will be available in clinical practice in the coming years, we pointed out an urgent need to better characterize hMPV infections, in order to better guide therapeutic management and use of forthcoming antiviral therapies. The present study aimed to describe the clinical, radiological and biological features, the course and outcomes of hMPV-associated lower respiratory tract infection in adult inpatients.

## 2 Methods

### 2.1 Study design and patient selection

We performed a prospective, observational, multicenter study in 24 secondary and seven tertiary care hospitals in France. From April 2018 to May 2019, all consecutive adult (≥18 years old) patients admitted to hospital with an acute lower respiratory tract infection and a respiratory multiplex Polymerase Chain Reaction (mPCR) test positive for hMPV within five days following hospital admission were included (for details regarding inclusion and exclusion criteria, see Electronic Supplementary Material, ESM). The mPCR used was the one available in the patient inclusion center, and may, therefore, differed between included patients (Table S1).

### 2.2 Data collection

For each recruited patient, pre-defined clinical, biological, microbiological and radiological data were collected by the investigator on an online electronic case report form (for details and definitions, see ESM and Table S2). Recruited patients were followed until hospital discharge (or until 60 days post-inclusion if the patient was still hospitalized).

### 2.3 Patient classification

Patients were classified according to the clinical diagnosis, determined by investigators for each patient using a six-class classification. The six mutually-exclusive classes of clinical diagnosis were: i) pneumonia, ii) bronchitis, iii) acute exacerbation of chronic obstructive pulmonary disease (COPD), iv) exacerbation of interstitial lung disease, v) pulmonary edema, and vi) other (for details regarding definitions used for patient classification, see ESM).

### 2.4 End points

The primary end point named “complicated course” was a composite of the death during hospital stay (censored at day-60) and/or the need for invasive mechanical ventilation.

### 2.5 Statistical analysis

Continuous data were expressed as median [first and third quartiles] and were compared using the non-parametric Mann-Whitney test. Categorical variables were expressed as numbers (percentages) and were evaluated using the Fischer exact test. P values less than 0.05 were considered significant. Comparisons focused on demographics, comorbidities, disease history, clinical, biological, microbiological and radiological findings, and hospital course, according to the primary composite end point. Characteristics of patients were also compared between those with a clinical diagnosis of pneumonia (pneumonia patients) versus those with another clinical diagnosis among the five other classes (non-pneumonia patients).

A multivariable logistic regression model was built to identify variables independently associated with the primary composite end point. All variables deemed clinically relevant were included without univariable screening. For this analysis, missing data were imputed using multiple imputation by chained equations: 30 datasets were imputed by predictive mean matching and logistic regression for continuous and binary variables, respectively, and estimates obtained from these datasets were pooled using Rubin's rule. All tests were two-tailed at the 0.05 significance threshold.

Analyses were performed using the R software version 4.0.1 (R Foundation for Statistical Computing, Vienna, Austria).

### 2.6 Ethical considerations

This study was approved by the institutional review board of the French Society of Respiratory Diseases (Reference CEPRO 2017-037), and verbal informed consent was obtained from all the participants before enrollment, according to the French regulations.

## 3 Results

### 3.1 Initial presentation

During the 13-month study period, 208 patients were included in the 31 participating centers (Fig. S1). Their clinical characteristics are depicted in Table 1. Median age was 74 years [62–84] and 97 (47 %) were males. A large proportion (90 %) had at least one comorbidity, and near a third of patients (31 %) had at least one immunosuppressive condition. The median time from symptom onset to hospital admission was three [2–7] days. The two most frequent symptoms were dyspnea (86 %) and cough (85 %); the triad fever-cough-dyspnea was observed in 42 % of patients. Clinical diagnosis was available in only 199 patients. The three most frequent were pneumonia (40 %), acute bronchitis (20 %) and acute exacerbation of chronic obstructive pulmonary disease (16 %). Radiological and laboratory findings at hospital admission are depicted in Table 2. Only 16 (8 %) patients had bilateral opacities on chest X-ray. In the 52 patients who had a chest computerized tomography (chest-CT) in the 96 h following hospital admission, the two most frequent abnormalities were ground glass opacity (41 %) and centrilobular micronodules (41 %). Leukocytosis (>10 G/L) was present in 31 % of the patients, lymphopenia (<1.5 G/L) in 78 % and thrombocytopenia (<150 G/L) in 24 %. A viral coinfection was diagnosed in 26 (13 %) patients, with rhinovirus (5 %) and RSV (5 %) as predominant species (Table 3). A bacterial coinfection was diagnosed in 61 (29 %) patients, with mixed flora (16 %) and enterobacteria (5 %) as predominant documentations. Of note, the frequency of bacterial coinfection did not differ between pneumonia patients and non-pneumonia patients. Among patients without coinfection (“pure” hMPV infection), the clinical diagnosis was pneumonia in 42 % of cases.

**Table 1:** Clinical characteristics of adult inpatients with hMPV-associated lower respiratory tract infection.

		Clinical diagnosis			Complicated course		
	All patients	Non-pneumonia	Pneumonia		No	Yes	
	N = 208	N = 115	N = 84	p-value	N = 165	N = 37	p-value
Age (years)	74 [62–84]	73 [62–84]	75 [61–84]	0.78	73 [62–84]	79 [64–84]	0.30
<30 years	3 (1.4)	1 (0.9)	2 (2.4)		2 (1.2)	1 (2.7)	
30–60 years	41 (19.7)	21 (18.3)	18 (21.4)		35 (21.2)	5 (13.5)	
>60 years	164 (78.8)	93 (80.9)	64 (76.2)		128 (77.6)	31 (83.8)	
Male sex	97 (46.6)	52 (45.2)	39 (46.4)	0.89	76 (46.1)	17 (45.9)	1
Former smoker	89 (47.1)	72 (47.7)	15 (45.5)	1	51 (48.1)	34 (43.6)	1
Current smoker	18 (9.5)	15 (9.9)	3 (9.1)	0.55	10 (9.4)	8 (10.3)	0.85
Time from symptom onset to hospital admission (days)	3 [2–7]	3 [2–7]	3 [1–6]	0.16	3 [2–7]	2 [1–4]	0.33
Body temperature (°C) at hospital admission				<0.01			0.72
<37.5	44 (22.8)	30 (28.6)	12 (14.5)		37 (24.2)	6 (17.6)	
37.5–39.0	72 (37.3)	45 (42.9)	25 (30.1)		57 (37.3)	13 (38.2)	
>39	77 (39.9)	30 (28.6)	46 (55.4)		59 (38.6)	15 (44.1)	
Symptoms and clinical signs at hospital admission^a^							
Nasal congestion	64 (34.4)	41 (41.0)	20 (24.7)	0.03	51 (34.7)	10 (30.3)	0.69
Headache	32 (17.3)	18 (18.2)	13 (16.0)	0.84	26 (17.8)	5 (15.2)	0.81
Cough	166 (84.7)	93 (85.3)	68 (82.9)	0.69	137 (87.8)	24 (70.6)	0.02
Sore throat	23 (12.6)	13 (13.4)	9 (11.1)	0.82	16 (11.1)	7 (21.2)	0.15
Sputum production	105 (53.3)	62 (56.4)	42 (51.2)	0.56	92 (58.6)	11 (32.4)	0.01
Asthenia	104 (54.7)	59 (56.7)	43 (52.4)	0.66	82 (54.7)	18 (52.9)	0.85
Dyspnoea	167 (85.6)	91 (84.3)	71 (86.6)	0.69	132 (85.7)	30 (85.7)	1
Nausea/vomiting	25 (13.2)	13 (12.6)	12 (14.6)	0.83	18 (12.0)	6 (17.6)	0.4
Diarrhea	21 (11.0)	9 (8.7)	11 (13.4)	0.35	17 (11.3)	4 (11.8)	1
Muscle ache	13 (7.1)	8 (8.2)	5 (6.2)	0.77	10 (7.0)	3 (9.1)	0.71
Wheezing	72 (36.9)	40 (36.7)	30 (37.0)	1	59 (37.8)	10 (30.3)	0.55
Crackles	106 (54.4)	55 (50.5)	47 (58.0)	0.31	79 (50.6)	23 (69.7)	0.06
Comorbidities^a^							
At least one	187 (89.9)	105 (91.3)	77 (91.7)	1	150 (90.9)	31 (83.8)	0.23
COPD	22 (10.9)	19 (17.0)	2 (2.4)	<0.01	17 (10.6)	4 (11.4)	1
Non-COPD lung disease	66 (31.7)	43 (37.4)	21 (25.0)	0.07	56 (33.9)	8 (21.6)	0.17
Diabetes	22 (10.9)	10 (8.9)	12 (14.3)	0.26	15 (9.4)	6 (17.1)	0.23
Hypertension	105 (52.2)	57 (50.9)	44 (52.4)	0.89	79 (49.4)	21 (60.0)	0.27
Coronary heart disease	37 (18.5)	23 (20.7)	13 (15.5)	0.46	29 (18.2)	8 (22.9)	0.63
Cerebrovascular disease	20 (10.0)	10 (8.9)	9 (10.7)	0.81	17 (10.6)	3 (8.6)	1
Chronic renal disease	6 (3.0)	2 (1.8)	4 (4.8)	0.41	5 (3.1)	1 (2.9)	1
Chronic immunosuppression^a^							
At least one	64 (30.8)	30 (26.1)	32 (38)	0.09	49 (29.7)	11 (29.7)	1
HIV infection	3 (1.4)	2 (1.8)	1 (1.2)	1	2 (1.2)	1 (2.7)	0.45
Neutropenia (<0.5 G/L)	1 (0.5)	1 (0.9)	0	1	0	1 (2.7)	0.18
Corticosteroid therapy	26 (12.5)	12	13 (15.4)	1	18 (10.9)	4 (11.4)	1
Immunosuppressive therapy	47 (22.6)	20 (17.4)	25 (29.8)	0.58	35 (21.2)	8 (21.6)	1
Solid organ transplantation	15 (7.2)	6 (5.2)	8 (9.5)	0.28	13 (7.9)	2 (5.4)	1
Bone marrow transplantation	10 (4.8)	4 (3.5)	6 (7.1)	0.33	9 (5.5)	1 (2.7)	1
Hemopathy/cancer	25 (12.0)	13 (11.3)	11 (13.1)	0.83	17 (10.3)	6 (16.2)	0.39

Data are presented as median [first through third quartiles] or number (%). Patients were classified according to the clinical diagnosis, which was available in 199 patients. Non-pneumonia diagnosis included bronchitis, acute exacerbation of COPD, exacerbation of interstitial lung disease, pulmonary edema, and other diagnosis. The composite criterion of “complicated course”, defined by death in hospital or at D60 or the need for invasive mechanical ventilation, was available for 202 patients.

Abbreviations: COPD, Chronic Obstructive Pulmonary Disease; HIV, Human Immunodeficiency Virus.

a multiple responses were possible.

**Table 2:** Radiological and laboratory findings in adult inpatients with hMPV-associated lower respiratory tract infection.

		Clinical diagnosis		p-value	Complicated course		p-value
	All patients	Non-pneumonia	Pneumonia		No	Yes	
	N = 208	N = 115	N = 84		N = 165	N = 37	
Chest X-ray							
Unilateral opacity	105 (50.4)	44 (38.3)	59 (70.2)	<0.01	80 (48.5)	22 (59.5)	0.28
Bilateral opacity	16 (7.7)	8 (7.0)	8 (9.5)	0.60	14 (8.5)	2 (5.4)	0.74
Pleural effusion	9 (5.1)	6 (6.0)	3 (4.2)	0.74	9 (6.3)	0 (0.0)	0.36
Chest CT							
Available Chest CT	52 (25.0)	21 (18.3)	27 (32.1)	0.03	35 (21.2)	12 (32.4)	0.20
Ground glass opacity	26 (41.3)	8 (29.6)	14 (43.8)	0.06	16 (35.6)	6 (46.2)	0.53
Alveolar condensation	23 (36.5)	7 (25.9)	17 (53.1)	0.29	15 (33.3)	8 (61.5)	0.11
Micronodule	26 (41.3)	14 (51.9)	11 (34.4)	0.20	18 (40.0)	7 (53.8)	0.53
Nodule	15 (23.8)	6 (22.2)	7 (21.9)	1	8 (17.8)	4 (30.8)	0.44
Pleural effusion	7 (11.1)	4 (14.8)	3 (9.4)	0.69	3 (6.7)	4 (30.8)	0.04
Laboratory findings							
Leukocyte count	8 [6–12]	8 [6–12]	8 [7–12]	0.93	8 [7–12]	9 [6–13]	0.90
Platelet count	203 [152–273]	210 [154–286]	196 [151–256]	0.24	208 [161–278]	186 [141–249]	0.25
CRP	46 [23–119]	41 [22–108]	66 [28–160]	0.07	43 [23–113]	98 [31–192]	0.05
Urea	8 [5–11]	7 [5–10]	9 [6–12]	0.05	7 [5–10]	9 [5–15]	0.13
Sodium	138 [135–140]	138 [136–140]	138 [134–139]	0.32	138 [136–140]	137 [133–139]	0.02

Data are presented as median [first through third quartiles] or number (%). Patients were classified according to the clinical diagnosis, which was available in 199 patients. Non-pneumonia diagnosis included bronchitis, acute exacerbation of COPD, exacerbation of interstitial lung disease, pulmonary edema, and other diagnosis. The composite criterion of “complicated course”, defined by death in hospital or at D60 or the need for invasive mechanical ventilation, was available for 202 patients. Leukocyte and platelet counts are expressed in 10^9^/L, CRP in mg/L, urea and sodium in mmol/L.

Abbreviations: CT, Computed Tomography; CRP, C Reactive Protein.

**Table 3:** Microbiological finding in adult inpatients with hMPV-associated lower respiratory tract infection.

		Final diagnosis		p-value	Complicated course		p-value
	All patients	Non-pneumonia	Pneumonia		No	Yes	
	N = 208	N = 115	N = 84		N = 165	N = 37	
Any virus	26 (12.5)	15 (13.0)	11 (13.1)	1	20 (12.1)	4 (10.8)	1
Rhinovirus	10 (4.8)	7 (6.1)	3 (3.6)	0.52	1 (0.6)	9 (24.3)	0.69
Respiratory syncitial virus	10 (4.8)	4 (3.5)	0 (0)	0.33	2 (1.2)	7 (18.9)	0.67
Influenza virus	4 (1.9)	4 (3.5)	6 (7.1)	0.14	0 (0)	3 (8.1)	1
Bocavirus	3 (1.4)	3 (2.6)	2 (2.4)	0.57	1 (0,6)	2 (5.4)	0.33
Coronavirus	3 (1.4)	1 (0.9)	0 (0)	0.27	0 (0)	3 (8.1)	1
Adenovirus	2 (1)	1 (0.9)	1 (1.2)	1	0 (0)	2 (5.4)	1
Parainfluenza virus	1 (0.5)	1 (0.9)	0 (0)	1	0 (0)	1 (2.7)	1
Any bacteria	61 (29.3)	33 (28.7)	24 (28.6)	0.16	45 (27.3)	13 (35.1)	1
Mixed flora	33 (15.9)	19 (16.5)	10 (11.9)	0.42	24 (14.5)	7 (18.9)	0.46
*Staphylococcus aureus*	8 (3.8)	5 (4.3)	3 (3.6)	1	5 (3)	3 (8.1)	0.16
Streptococcus pneumoniae	7 (3.4)	3 (2.6)	4 (4.8)	0.46	5 (3)	1 (2.7)	1
Other Streptococcus	2 (1)	0 (0)	1 (1.2)	0.42	1 (0.6)	1 (2.7)	0.33
Haemophilus influenzae	6 (2.9)	2 (1.7)	4 (4.8)	0.24	5 (3)	1 (2.7)	1
Enterobacteriaceae	11 (5.3)	5 (4.3)	6 (7.1)	0.53	10 (6.1)	1 (2.7)	0.69
*Pseudomonas aeruginosa*	4 (1.9)	3 (2.6)	1 (1.2)	0.64	3 (1.8)	1 (2.7)	0.56
Mycoplasma pneumoniae	6 (2.9)	1 (0.9)	5 (6)	0.08	5 (3)	1 (2.7)	1
Chlamydia pneumoniae	2 (1)	1 (0.9)	1 (1.2)	1	1 (0.6)	1 (2.7)	0.33
*Legionella pneumophila*	2 (1)	2 (1.7)	0 (0)	0.51	2 (1.2)	0 (0)	1
Bordetella pertussis	0 (0)	1 (0.9)	0 (0)	1	1 (0.6)	0 (0)	1

Data are presented as median [first through third quartiles] or number (%). Patients were classified according to the clinical diagnosis, which was available in 199 patients. Non-pneumonia diagnosis included bronchitis, acute exacerbation of COPD, exacerbation of interstitial lung disease, pulmonary edema, and other diagnosis. The composite criterion of “complicated course”, defined by death in hospital or at D60 or the need for invasive mechanical ventilation, was available for 202 patients.

### 3.2 Hospital course and outcomes

Hospital course and outcomes are depicted in Table 4. The primary composite end point was assessable in 202 patients. Among them, a complicated course was observed in 37 (18 %) patients, without difference between pneumonia patients and non-pneumonia patients (16 % vs. 19 %, p = 0.61) (Table S3). The multivariable analysis identified tachycardia and alteration of consciousness (Glasgow <15) as independently associated with a complicated course (Table S4). A majority of patients received empirical antibiotic therapy at hospital admission, but with a larger proportion in pneumonia patients (88 % vs. 76 %, p = 0.01). The median length of hospital stay was nine days [6–14]. Near a third of patients were admitted to the ICU, with a median length of ICU stay of six [3–9] days (Table S5). Among them, 38 % required invasive mechanical ventilation for a median duration of six [4–12] days. Hospital death occurred in ten (12 %) pneumonia patients, as compared to seven (6 %) non-pneumonia patients (p = 0.19). Interestingly, a complicated course was more frequently observed in pneumonia patients with bacterial coinfection (8/24, 33 %) as compared to those without bacterial coinfection (5/60, 8 %) (p = 0.02).

**Table 4:** Hospital course and outcomes in adult inpatients with hMPV-associated lower respiratory tract infection.

		Final diagnosis		p-value	Complicated course		p-value
	All patients	Non-pneumonia	Pneumonia		No	Yes	
	N = 208	N = 115	N = 84		N = 165	N = 37	
Antibiotics	159 (80.7)	84 (75.7)	73 (88.0)	0.04	124 (77.5)	31 (96.9)	0.01
Antiviral therapy	30 (15.1)	13 (11.4)	16 (19.5)	0.15	19 (11.7)	9 (27.3)	0.03
Systemic glucocorticoids	53 (27.0)	34 (29.5)	19 (22.6)	0.26	37 (23.3)	14 (42.4)	0.03
Admission to the ICU	60 (28.8)	31 (27.0)	27 (32.1)	0.44	33 (20.0)	26 (70.3)	<0.01
Non-invasive ventilation	27 (12.9)	18 (15.7)	9 (10.7)	0.03	16 (9.7)	11 (29.7)	<0.01
Invasive mechanical ventilation	23 (11.1)	14 (12.2)	8 (9.5)	0.65	–	23 (62.2)	<0.01
ARDS	14 (6.7)	5 (4.3)	9 (10.7)	0.49	3 (1.8)	11 (29.7)	<0.01
Length of hospital stay (days)	9 [6–14]	11 [7–17]	8 [5–10]	0.03	9 [6–13]	29 [19–34]	<0.01
Death	17 (8.3)	7 (6.1)	10 (12.0)	0.20	–	17 (45.9)	NA

Data are presented as median [first through third quartiles] or number (%). Patients were classified according to the clinical diagnosis, which was available in 199 patients. Non-pneumonia diagnosis included bronchitis, acute exacerbation of COPD, exacerbation of interstitial lung disease, pulmonary edema, and other diagnosis. The composite criterion of “complicated course”, defined by death in hospital or at D60 or the need for invasive mechanical ventilation, was available for 202 patients.

Abbreviations: ARDS, Acute Respiratory Distress Syndrome; ICU, Intensive Care Unit.

## 4 Discussion

This study investigated the clinical spectrum and outcomes of hMPV-associated lower respiratory tract infection in hospitalized adults. Patients were mostly elderly people with comorbid conditions, with a clinical diagnosis of pneumonia or acute bronchitis in two third of them and a bacterial coinfection documented in 29 % of patients. Among patients without coinfection (“pure” hMPV infection), the clinical diagnosis was pneumonia in 42 % of cases. In the 52 patients who had a chest-CT, the most frequent abnormality was ground glass opacity (41 %). A complicated course, defined as the death during hospital stay and/or the need for invasive mechanical ventilation, was observed in 18 % of patients, but up to 33 % of pneumonia patients with a bacterial coinfection.

In this cohort, we observed a high frequency of dyspnea and cough, in line with previous findings in hMPV-infected inpatients (Table 5) [2,3,6,11]. These symptoms are not specific of hMPV, since similar observations have been reported with other respiratory viruses [12]. Our population displayed a high rate of comorbid conditions and an advanced age. These demographical features, which have also been pointed out in RSV infection, differ from influenza-infected inpatients [2,3,6,11,[13], [14], [15], [16], [17], [18]]. Paraclinical data revealed ground glass opacities and micronodules as predominant CT-scan abnormalities, in line with a previous report in hematological patients [19].

**Table 5:** Overview of studies reporting cohorts of adult inpatients affected of hMPV-associated lower respiratory tract infections.

	Hamlin et al.	Walsh et al.^a^	Hasvold et al.	Loubet et al.	Our cohort
	2005	2008	2016	2020	
	Clin Infect Dis	Arch Intern Med	J Crit Care	Clin Microbiol Infect	
Design	Prospective	Prospective	Retrospective	Retrospective	Prospective
Patients	6	91	128	90	208
Median age	64	NA	55	78	74
Comorbidities	6 (100 %)	NA	89 (70 %)	73 (81 %)	187 (90 %)
Chronic immunosuppression	NA	NA	NA	15 (17 %)	67 (32 %)
Most common symptom	Cough (100 %)	Dyspnea (98 %)	NA	Fever (88 %)	Dyspnea (86 %)
Second most common symptom	Dyspnea (83 %)	Cough (94 %)	NA	Dyspnea (84 %)	Cough (85 %)
Most common lung CT-scan abnormality	NA	NA	NA	NA	GGO (41 %)^b^
Virus-virus co-infection	1 (17 %)	NA	1 (1 %)	NA	26 (13 %)
Virus-bacterial co-infection	1 (17 %)	1 (2 %) c	NA	NA	61 (29 %)
Final diagnosis of pneumonia	70 %	25 %	NA	36 %	42 %
Admission in ICU	NA	12 (13 %)	40 (31 %)	15 (17 %)	60 (29 %)
Invasive mechanical ventilation	0	11 (11 %)	22 (17 %)	NA	23 (11 %)
Hospital stay duration, days	10	9	5	7	9
Death	0	6 (7 %)	10 (8 %)	4 (4 %)	17 (8 %)

Data are presented as median [first through third quartiles] or number (%) unless otherwise stated.

a Only the cohort of hospitalized patients.

b Among the 52 patients who had a lung CT-scan.

c Among the 31 patients who had a bacteriological examination of a respiratory sample.

A bacterial documentation was obtained in 29 % of cases, a rate slightly higher than previously reported in hMPV-infected hospitalized adults but in the range of values, from 18 to 31 %, reported in RSV infection [2,3,6,11]. The predominant bacterial documentation was mixed respiratory flora, in line with recent findings showing this polymicrobial documentation as predominant in community-acquired pneumonia (CAP) inpatients with viral-bacterial coinfection [20]. Additionally, we observed that gram-negative bacilli (GNB), including Enterobacteriaceae and *P. aeruginosa*, accounted for 7 % of bacterial documentation in the whole cohort and 8 % in the subgroup of patients with a clinical diagnosis of pneumonia. These rates were slightly higher than those reported in cohorts of hospitalized patients with influenza infection, but in line with recent findings by Coussement et al. who reported a rate of GNB-related coinfection of 8 % in 309 patients with severe RSV infection [[21], [22], [23]]. This observation may be related to the advanced age of our population. Indeed, GNB have been shown predominant in cohorts of older patients with community-acquired pneumonia, probably linked to an age-dependent increased risk of aspiration [[24], [25], [26]].

Viral-bacterial coinfection has been shown to be associated with poor outcome in hospitalized adults with moderate-to-severe CAP [[27], [28], [29]]. In line with these results, we observed in the subpopulation of pneumonia patients a more complicated course in patients with a bacterial coinfection as compared to those without. This finding is original since none of the previous studies in hMPV-infected patients addressed this specific point, while at least one study reported similar results in RSV infection [18]. Admission to the ICU and hospital death occurred in 29 % and 8 % of our patients, respectively. Both these values were in the upper range (Table 5) of values reported in previous cohorts of hMPV-infected patients [2,3,6,11].

Multivariable analysis identified tachycardia and alteration of consciousness as independently associated with a complicated course. Interestingly, none of these parameters was related to respiratory failure. These findings should encourage clinicians to carefully take extrapulmonary clinical manifestations into account when caring for adult patients with lower respiratory tract infection. Indeed, the high burden of non-pulmonary signs and symptoms has been described for a long time in elderly and immunocompromised patients with either CAP or respiratory virus-associated infection [30,31]. Finally, these predictors of poor outcome may be helpful for clinicians in order to identify at-risk patients and guide the use of forthcoming drugs targeting hMPV.

Our study has several limitations. First, we did not control the diagnosis of hMPV infection. The decision to perform a respiratory mPCR was at the discretion of physicians, based on their clinical suspicion of lower respiratory tract infection; this might suggest a confounding of indication. However, recent results by our group have shown that mPCR tests were widely used by clinicians worldwide in a syndromic approach to the microbiological diagnosis in hospitalized patients [32]. Second, we did not control paraclinical investigations. A lung CT-scan was performed in only a quarter of patients, preventing any detailed analysis of CT-scan patterns in the whole study population. Some microbiological tests were only occasionally performed because the bacteriological work-up was at the attending clinician's discretion. It meant that some bacterial coinfections could have been missed, so the proportion of patients with coinfection could have been underestimated. Additionally, we did not collect post-infection clinical data such as pulmonary function test, which would have highlighted the long-term consequences of hMPV infection in survivors. Third, mPCR test differed from one center to another, but all the centers used tests with CE marking certification that guarantee high safety and level of performance. Fourth, we considered that any hMPV documentation within airways affirmed the diagnosis of hMPV infection, regardless of the site of sampling (upper or lower respiratory tract). This assumption was supported by the natural history of Pneumoviridae-related infections, which are known to invade the whole respiratory tract [33]. Fifth, we chose a primary composite end point. Indeed, considering the predictable low hospital mortality, we did not choose hospital death as primary end point, because the low frequency of the event would have favored the absence of difference between groups.

In conclusion, hMPV-associated lower respiratory tract infections in adult inpatients mostly involved elderly people with pre-existing conditions. The most frequent clinical diagnosis was pneumonia. A bacterial coinfection was documented in 29 % of patients. The need for mechanical ventilation and/or the death were observed in 18 % of patients, but up to a third of pneumonia patients with a bacterial coinfection. Our data highlight the crucial need for development of antiviral drugs targeting hMPV.

## Ethics to approval and consent to participate

This study was approved by the institutional review board of the French Society of Respiratory Diseases (Reference CEPRO 2017-037) according to the French regulations. The board waived the need for signing consent for patients included in the study.

## Consent for publication

Not applicable.

## Availability of supporting data

Data and materials supporting the findings of this study can be entirely shared if asking.

## Funding

None.

## Data availability statement

The data needed to evaluate the conclusions in the paper are present in the paper or the ESM. Raw data are available upon request from the corresponding authors under a material/data transfer agreement.

## CRediT authorship contribution statement

**Quentin Philippot:** Writing – review & editing, Writing – original draft, Investigation, Conceptualization. **Blandine Rammaert:** Investigation. **Gaëlle Dauriat:** Investigation. **Cédric Daubin:** Investigation. **Frédéric Schlemmer:** Investigation. **Adrien Costantini:** Investigation. **Yacine Tandjaoui-Lambiotte:** Investigation. **Mathilde Neuville:** Investigation. **Emmanuelle Desrochettes:** Investigation. **Alexis Ferré:** Investigation. **Laetitia Bodet Contentin:** Investigation. **François-Xavier Lescure:** Investigation. **Bruno Megarbane:** Investigation. **Antoine Belle:** Investigation. **Jean Dellamonica:** Investigation. **Sylvain Jaffuel:** Investigation. **Jean-Luc Meynard:** Investigation. **Jonathan Messika:** Investigation. **Nicolas Lau:** Investigation. **Nicolas Terzi:** Investigation. **Isabelle Runge:** Investigation. **Olivier Sanchez:** Investigation. **Benjamin Zuber:** Investigation. **Emmanuel Guerot:** Investigation. **Anahita Rouze:** Investigation. **Patricia Pavese:** Investigation. **François Bénézit:** Investigation. **Jean-Pierre Quenot:** Investigation. **Xavier Souloy:** Investigation. **Anne Lyse Fanton:** Investigation. **David Boutoille:** Investigation. **Vincent Bunel:** Investigation. **Astrid Vabret:** Investigation. **Jacques Gaillat:** Investigation. **Anne Bergeron:** Supervision, Conceptualization. **Nathanaël Lapidus:** Formal analysis. **Muriel Fartoukh:** Investigation. **Guillaume Voiriot:** Writing – review & editing, Writing – original draft, Investigation, Funding acquisition.

## Declaration of competing interest

The authors declare the following financial interests/personal relationships which may be considered as potential competing interests:Astrid VABRET disclosed payment or honoraria for lectures, presentations and support for attending meetings and/or travel from ANOFI, 10.13039/501100003103ASTRA ZENECA, MODERNA, and participation on a Data Safety Monitoring Board from SANOFI, 10.13039/100030732MSD, MODERNA and GSK. Frederic Schlemmer disclosed consulting fees from 10.13039/100004319Pfizer, payment or honoraria for lectures, presentations from 10.13039/100005564Gilead, and participation on a Data Safety Monitoring Board from Boerhinger Ingelheim, 10.13039/100019719Chiesi, Elivie, 10.13039/100005564Gilead, 10.13039/100004330GlaxoSmithKline and Oxyvie. Anne Bergeron disclosed payment or honoraria for lectures, presentations and support for attending meetings and/or travel from Astra Zeneca and 10.13039/100004336Novartis, and participation on a Data Safety Monitoring Board from Enanta. Anahita Rouzé disclosed payment or honoraria for lectures, presentations and support for attending meetings and/or travel from 10.13039/100030732MSD, 10.13039/100005564Gilead, Mundipharma. Blandine RAMMAERT disclosed payment or honoraria for lectures, presentations from Gilead. Cedric Daubin disclosed support for attending meetings and/or travel from 10.13039/100005564Gilead. Nicolas Terzi disclosed payment or honoraria for lectures, presentations from 10.13039/100004319Pfizer and Boehringher Inghelheim, and support for attending meetings and/or travel from 10.13039/100005564Gilead. Quentin Philippot disclosed payment or honoraria for lectures, presentations, speakers bureaus, manuscript writing or educational events from 10.13039/100005564Gilead and Elsevier, and grants or contracts from APHP, 10.13039/501100017007ARS Ile de France and 10.13039/501100001677Inserm 10.13039/501100007492Fondation Bettencourt Schueller, and support for the present manuscript from SOS Oxygen. Other authors did not disclose competing financial interests or personal relationships that could have appeared to influence the work reported in this paper.

## List of abbreviations

CAP: community-acquired pneumonia
chest-CT: chest computerized tomography
COPD: chronic obstructive pulmonary disease
ESM: electronic supplementary material
GNB: gram-negative bacilli
hMPV: Human metapneumovirus
ICU: intensive care unit
mPCR: multiplex polymerase chain reaction
RSV: respiratory syncytial virus
