# Supplementary material for: Human metapneumovirus infection is associated with a substantial morbidity and mortality burden in adult inpatients
Source: Heliyon. 2024 Jun 18;10(13):e33231. doi: 10.1016/j.heliyon.2024.e33231 (PMC11259828; doi:10.1016/j.heliyon.2024.e33231)
Supplement: Multimedia component 1 [file mmc1.docx]

**ELECTRONIC SUPPLEMENTAL MATERIAL**

**Inclusion and exclusion criteria**

A lower respiratory tract infection was defined by the presence of at least two of the following criteria: fever, cough, sputum production, signs of respiratory distress, thoracic pain, dyspnea, crackles, need for oxygen, and need for mechanical ventilation. Respiratory mPCR may be performed either in nasopharyngeal (NP) swabs or in lower respiratory tract (LRT) specimen (endotracheal aspirate or bronchoalveolar lavage). Different commercially available mPCR tests have been used in the 24 participating centers. The only exclusion criteria was a previous inclusion in the present study.

**Definitions**

Chronic immunosuppression was defined by at least one of the following conditions: splenectomy, neutropenia (<0.5 G/L), HIV infection, long-term steroid therapy, other long-term immunosuppressive therapy, solid organ or bone marrow transplantation, and malignant hemopathy or cancer. Chest computerized tomography (chest-CT) was considered only if performed within four days following hospital admission. For patients admitted to the intensive care unit (ICU), the Simplified Acute Physiology Score II (SAPS II) was calculated based of the worse values recorded during the first 24 hours of the ICU stay.

**Definitions used for patient classification**

Pneumonia was defined as the association of i) at least two signs of systemic inflammatory response; ii) a new infiltrate evidenced on the chest radiograph (or chest-CT); and iii) a recent onset of at least two of the following signs of pneumonia: cough, sputum production, auscultatory findings consistent with pneumonia, and fever.

Acute bronchitis was defined as the association of i) at least two signs of systemic inflammatory response; ii) the absence of new infiltrate evidenced in the chest radiograph (or chest-CT); and iii) a recent onset of at least two of the following signs of bronchitis: cough, sputum production, auscultatory findings consistent with bronchitis, and fever.

Acute exacerbation of chronic obstructive pulmonary disease (COPD) was defined as an acute worsening of respiratory symptoms compared to basal condition that is sufficient to require a change in treatment.

**Microbiological investigations**

A non-hMPV viral documentation within airways was always considered as a virus-virus coinfection, regardless of the type of respiratory sample.

The decision to perform microbiological investigations for non-viral pathogens was up to the discretion of clinicians. Respiratory tract specimens underwent Gram staining and quantitative culture for bacterial pathogen, urine antigen testing of *Streptococcus pneumoniae* and *Legionella pneumophila* used the BinaxNOW kits (Alere®). A bacterium was considered as a copathogen if this bacterium fulfilled at least one criteria among the followings: i) *Streptococcus pneumoniae* or *Staphylococcus aureus* or *Legionella pneumophila*, whatever the test and level of positivity; ii) identified in pleural fluid or blood; iii) *Chlamydia pneumoniae* or *Mycoplasma pneumoniae* identified with Ig antibodies testing; iv) *Bordetella pertussis* or *Chlamydiae pneumoniae* or *Mycoplasma pneumoniae* identified by mPCR; v) identified within a sputum specimen with good-quality criteria (leucocyte > 25/field and epithelial cells < 10/field) and ≥ 10^6^ colony-forming units/mL); vi) identified within bronchoalveolar lavage fluid at ≥ 10^4^ colony-forming units/mL or protected distal sample at ≥ 10^3^ colony-forming units/mL, or bronchial aspirate at ≥ 10^5^ colony-forming units/mL.

**Table S1.** Name and company of the mPCR tests used for the hMPV infection diagnosis.

| **Name, Company** | **N (%)** |
| --- | --- |
| BioFire® FilmArray®, Biomérieux | 120 (58) |
| Anyplex^TM^, Seegene | 46 (22) |
| GenMark ePlex®, Roche Diagnostics | 20 (10) |
| RespiFinder®, Pathofinder | 4 (2) |
| Other | 11 (5) |
| Unknown | 7 (3) |

**Table S2.** Pre-defined variables collected for each patient.

| **Background and comorbidities** | | |
| --- | --- | --- |
| - Age - Gender - Height - Weight - Smoke history - Asthma - COPD - Bronchiectasis - Chronic interstitial lung disease - Other chronic lung disease - Long-term oxygen therapy - Diabetes - Hypertension - Chronic heart failure NYHA III or IV - Coronary artery disease - Cerebral artery disease - Chronic dialysis - Cirrhosis Child Pugh B-C - WHO performance status | | - HIV infection - Splenectomy - Neutropenia (< 0.5 G/L) - Long-term corticosteroid therapy - Other long-term immunosuppressive therapy - Solid organ transplantation - Bone marrow transplantation - Malignant hemopathy or cancer - Chemotherapy in the last 30 days - Pregnancy - Inhaled corticosteroid use - Anti-pneumococcal vaccine in the last 5 years - Anti-flu vaccine - Institutionalization - Hospitalization for ≥ 2 days in the preceding 90 days - Antibiotics of > 24 hours in the preceding 90 days |
| **Symptoms and clinical signs at hospital admission** | | |
| - Date of symptom onset - Asthenia - Fever > 37.7°C - Dyspnoea - Cough - Sputum production - Nasal congestion - Thoracic pain - Rhinorrhoea - Sore throat - Sneezing - Otalgia - Conjunctivitis - Headache | | - Appetit loss - Nausea and/or vomiting - Diarrhoea - Muscle ache - Wheezing - Crackles - Squeaks - Rhonchi - Signs of respiratory distress - Skin rash - Minimal O_2_ pulse saturation - Maximal body temperature - Maximal respiratory rate - Maximal heart rate - Minimal Glasgow coma scale |
| **Biological (blood) work-up at hospital admission** | | |
| - Leucocyte count - Neutrophil count - Lymphocyte count - Platelet count - Haematocrit - Fibrinogen - Sodium - Creatinine - Urea - Creatine kinase - Brain natriuretic peptide | - Troponin - Procalcitonin - C reactive protein - Serum glutamooxaloacetate transferase (SGOT) - Serum glutamopyruvate transferase (SGPT) - Alkaline phosphatase (ALP) - Glycemia - Arterial pH - Arterial partial pressures of O_2_ and CO_2_ - Arterial HCO3- concentration - Arterial lactate concentration | |

**Table S2 (continued).**

| **Imaging findings at hospital admission** | |
| --- | --- |
| - Number of quadrants with abnormalities on chest X-Ray (performed within 24 hours following hospital admission) - Type of abnormality on chest X-ray - Type of abnormality on chest-CT (performed within 96 hours following hospital admission) | |
| **Microbiological work-up** | |
| - mPCR brand - Result of the respiratory bacterial work-up (sputum, tracheal aspiration, broncho-alveolar lavage) - Result of a second respiratory mPCR - Blood culture result | - Pneumococcal and legionella urinary antigen test results - Blood HSV PCR result - Blood CMV PCR result - Respiratory HSV PCR result - Respiratory CMV PCR result |
| **Severity score at ICU admission** | |
| - SAPS2 | |
| **Management during the hospital stay** | |
| - Corticosteroid treatment - High flow oxygen therapy - Mechanical ventilation - ARDS (Berlin definition) | - Prone ventilation - ECMO - Vasopressor support - Renal replacement therapy |
| **Follow-up** | |
| - Hospital acquired infection - Antibiotics - Antiviral drug - Clinical diagnosis | - Duration of ICU stay - Duration of hospital stay - Death |

Abbreviations: chest-CT, chest Computerized Tomography ; CMV, Cytomegalovirus ; COPD, Chronic Obstructive Pulmonary Disease ; ECMO: Extracorporeal Membrane ; HIV, Human Immunodeficiency Virus ; HSV, Human Simplex Virus ; ICU, Intensive Care Unit ; mPCR, multiplex Polymerase Chain Reaction ; RSV, Respiratory Syncytial Virus.

**Table S3.** Complicated course according to the clinical diagnosis in adult inpatients with hMPV-associated lower respiratory tract infection.

|  |  | Non-Pneumonia | | | | |
| --- | --- | --- | --- | --- | --- | --- |
|  | Pneumonia  N=84 | Acute  bronchitis  N=39 | Acute exacerbation  of COPD  N=32 | Exacerbation of interstitial lung disease  N=4 | Acute  pulmonary  edema  N=6 | Other  N=34 |
| Complicated course, n (%) | 13 (15.5) | 3 (7.7) | 8 (25.0) | 2 (50.0) | 2 (33.3) | 6 (17.6) |

Patients were classified according to the clinical diagnosis, which was available in 199 patients. The composite criterion of “complicated course”, defined by death in hospital or at D60 or the need for invasive mechanical ventilation, was available for 202 patients.

Abbreviation: COPD= Chronic Obstructive Pulmonary Disease.

**Table S4.** Multivariable analysis of the risk factors for complicated course in adult inpatients with hMPV-associated lower respiratory tract infection.

| Variable | OR [95% CI] | p-value |
| --- | --- | --- |
| Heart rate | 1.03 [1.01-1.05] | 0.001 |
| Oxygen saturation | 0.99 [0.93-1.04] | 0.621 |
| Dysnatremia | 1.91 [0.70-5.22] | 0.020 |
| Blood urea ≥ 10 mmol/L | 2.21 [0.86-5.69] | 0.100 |
| Platelet count < 150 G/L | 2.32 [0.85-6.38] | 0.101 |
| Leukocyte count < 4 G/L | 4.91 [0.90-26.9] | 0.067 |
| Glasgow Coma Scale = 15 | 0.24 [0 .07-0.79] | 0.019 |

The composite criterion of “complicated course”, defined by death in hospital or at D60 or the need for invasive mechanical ventilation, was available for 202 patients. Dysnatremia was defined as >145 mmol/l or < 135mmol/L. For continuous variables, the odds ratio represents the increase in odds for each unit (year for age, bpm for heart rate, percent for oxygen saturation).

Abbreviations: G/L, 10^9^/L ; OR, Odds ratio.

**Table S5.** Hospital course of adult inpatients with hMPV-associated lower respiratory tract infection admitted in the intensive care unit.

|  |  | Final diagnosis | |  | Complicated course | |  |
| --- | --- | --- | --- | --- | --- | --- | --- |
|  | All patients  N=60 | Non-pneumonia  N=31 | Pneumonia  N=27 | p-value | No  N=33 | Yes  N=26 | p-value |
| SAPS II score | 40 [30-45] | 36 [30-45] | 41 [33-43] | 0.44 | 33 [29-41] | 43 [38-55] | <0.01 |
| SOFA score | 4 [3-7] | 4 [3-6] | 4 [2-8] | 0.68 | 3 [2-4] | 6 [4-9] | <0.01 |
| ARDS | 14 (23.7) | 5 (16.1) | 9 (33.3) | 0.22 | 3 (9.1) | 11 (44.0) | <0.01 |
| Vasopressors | 14 (23.7) | 6 (19.4) | 8 (29.6) | 0.54 | 0 (0.0) | 14 (56.0) | <0.01 |
| RRT | 4 (6.8) | 1 (3.2) | 3 (11.1) | 0.33 | 0 (0.0) | 4 (16.0) | 0.03 |
| HFNO | 28 (47.5) | 15 (48.4) | 12 (44.4) | 0.80 | 15 (45.5) | 12 (48.0) | 1 |
| NIV | 27 (45.8) | 18 (58.1) | 9 (33.3) | 0.07 | 16 (48.5) | 11 (44.0) | 0.80 |
| MV | 23 (38.3) | 14 (45.2) | 8 (29.6) | 0.28 | 0 (0.0) | 23 (88.5) | <0.01 |

Data are presented as median [first through third quartiles] or number (%). Patients were classified according to the clinical diagnosis, which was available in 199 patients. Non-pneumonia diagnosis included bronchitis, acute exacerbation of COPD, exacerbation of interstitial lung disease, pulmonary edema, and other diagnosis. The composite criterion of “complicated course”, defined by death in hospital or at D60 or the need for invasive mechanical ventilation, was available for 202 patients.

Abbreviations: ARDS, Acute Respiratory Distress Syndrome ; HFNO, High-Flow Nasal Oxygen ; MV, invasive Mechanical Ventilation ; NIV, Non Invasive Ventilation ; RRT, Renal Replacement Therapy ; SAPS II, simplified Acute Physiology Score ; SOFA, Sepsis-related Organ Failure Assessment.


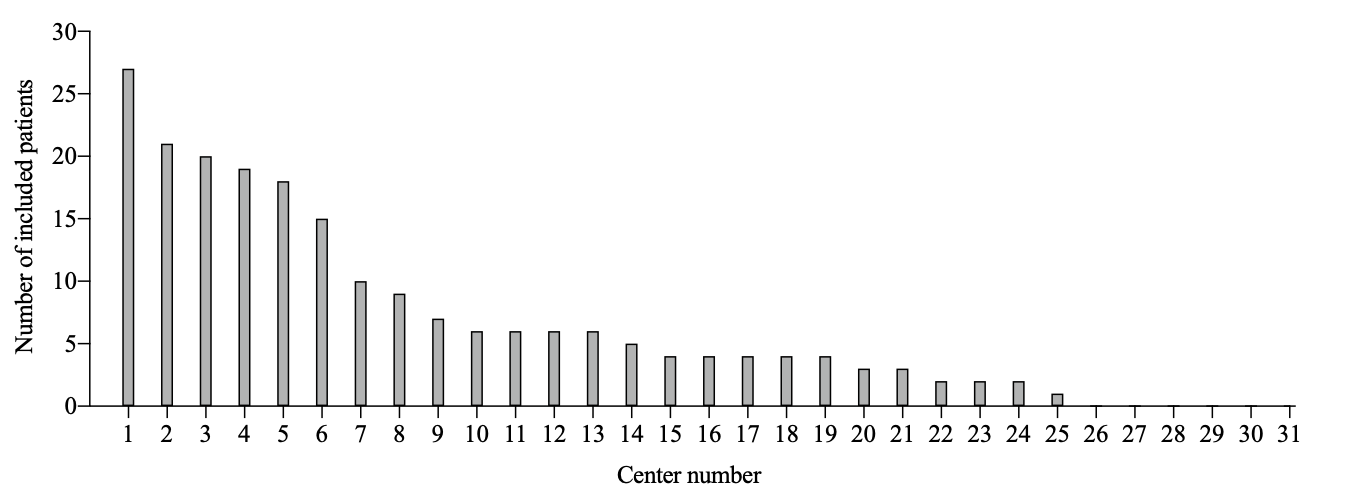


**Figure S1.** Number of patients included in each participating center.
